# Costs and Efficiency of Online and Offline Recruitment Methods: A Web-Based Cohort Study

**DOI:** 10.2196/jmir.6716

**Published:** 2017-03-01

**Authors:** Tina Christensen, Anders H Riis, Elizabeth E Hatch, Lauren A Wise, Marie G Nielsen, Kenneth J Rothman, Henrik Toft Sørensen, Ellen M Mikkelsen

**Affiliations:** ^1^ Aarhus University Hospital Department of Clinical Epidemiology Aarhus N Denmark; ^2^ Boston University School of Public Health Department of Epidemiology Boston, MA United States; ^3^ Aarhus University Research Unit for General Practice, Department of Public Health Aarhus C Denmark; ^4^ RTI Health Solutions Research Triangle Park, NC United States

**Keywords:** participant recruitment, Web-based study, costs per participant, Internet, advertising

## Abstract

**Background:**

The Internet is widely used to conduct research studies on health issues. Many different methods are used to recruit participants for such studies, but little is known about how various recruitment methods compare in terms of efficiency and costs.

**Objective:**

The aim of our study was to compare online and offline recruitment methods for Internet-based studies in terms of efficiency (number of recruited participants) and costs per participant.

**Methods:**

We employed several online and offline recruitment methods to enroll 18- to 45-year-old women in an Internet-based Danish prospective cohort study on fertility. Offline methods included press releases, posters, and flyers. Online methods comprised advertisements placed on five different websites, including Facebook and Netdoktor.dk. We defined seven categories of mutually exclusive recruitment methods and used electronic tracking via unique Uniform Resource Locator (URL) and self-reported data to identify the recruitment method for each participant. For each method, we calculated the average cost per participant and efficiency, that is, the total number of recruited participants.

**Results:**

We recruited 8252 study participants. Of these, 534 were excluded as they could not be assigned to a specific recruitment method. The final study population included 7724 participants, of whom 803 (10.4%) were recruited by offline methods, 3985 (51.6%) by online methods, 2382 (30.8%) by online methods not initiated by us, and 554 (7.2%) by other methods. Overall, the average cost per participant was €6.22 for online methods initiated by us versus €9.06 for offline methods. Costs per participant ranged from €2.74 to €105.53 for online methods and from €0 to €67.50 for offline methods. Lowest average costs per participant were for those recruited from Netdoktor.dk (€2.99) and from Facebook (€3.44).

**Conclusions:**

In our Internet-based cohort study, online recruitment methods were superior to offline methods in terms of efficiency (total number of participants enrolled). The average cost per recruited participant was also lower for online than for offline methods, although costs varied greatly among both online and offline recruitment methods. We observed a decrease in the efficiency of some online recruitment methods over time, suggesting that it may be optimal to adopt multiple online methods.

## Introduction

Recruiting participants for epidemiologic research is increasingly difficult as the number of projects competing for people’s attention increases and response rates decline [1]. In cohort studies, participant recruitment and data collection are associated with a heavy workload and high costs [2]. Widespread access to the Internet now offers an alternative strategy to recruit participants into cohort studies and to collect data. The Internet offers technical advantages in data collection that can reduce administrative procedures and improve data quality. Examples are incorporating skip patterns that avoid displaying irrelevant questions, building in internal consistency checks, and avoiding errors that occur during manual data entry [3]. Furthermore, the Internet is an effective tool to reach populations who are otherwise challenging to enroll because they have sensitive health concerns, including urinary incontinence, sexual health disorders, or mental health problems [4-6].

Pregnancy planners also constitute a hard-to-reach population as they do not typically announce their pregnancy intentions [7-10]. Among recruitment methods that have proven to be effective in enrolling participants are study-related media publicity [6,11], online advertisements [6,7,10,12-15], printed advertisements [6], and Web-based social groups [6,10].

Some studies have reported the costs of a single online recruitment method [14,16-19], and others have reported on costs of using several online methods [20,21], or both offline and online methods [6,10,12,22,23]. Nevertheless, little is known about how recruitment methods compare in terms of efficiency (number of recruited participants) and costs per participant [20]. Here, we compare the efficiency and costs per recruited participant of online and offline recruitment methods used to enroll women in a Danish cohort study of pregnancy planners that relied on the Internet for enrollment and data collection.

## Methods

### Setting

**Figure 1:**
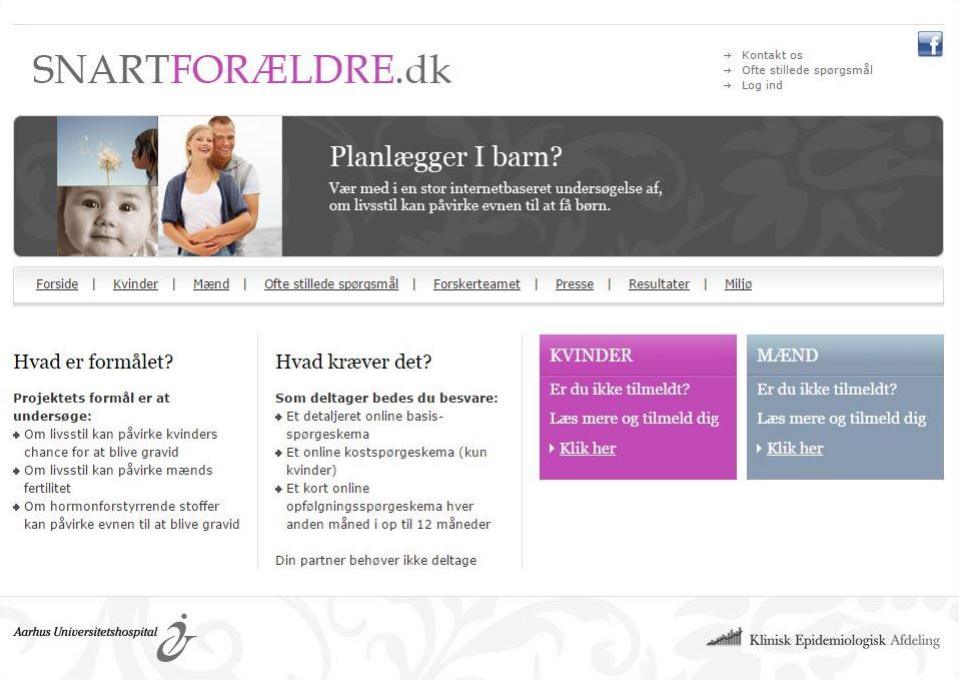
The frontpage of the SnartForaeldre.dk website.

The Snart-Gravid.dk (Soon-Pregnant) and the SnartForaeldre.dk (SoonParents) studies are related prospective cohort studies on lifestyle and fertility [8,9,24,25]. In both studies, participants enroll via the Internet and all data are collected by means of Web-based questionnaires. Snart-Gravid.dk was launched in June 2007. It was succeeded in August 2011 by SnartForaeldre.dk, which incorporates a dietary questionnaire and includes male partners (Figure 1). Recruitment for SnartForaeldre.dk is ongoing [26].

The study period for this paper, which focuses on female recruitment in both studies (in the following referred to as one study), is June 2007 through December 2013.

### Study Population

The recruitment area for Snart-Gravid.dk and SnartForaledre.dk covers all of Denmark (total population of 5.7 million on January 1, 2016, and 58,205 births in 2015) [27]. Eligible female participants have a Danish civil registration number, are aged 18-45 years old (18-40 years in Snart-Gravid.dk), in a stable relationship with a male partner, and attempting to become pregnant. To enroll, participants have to (1) visit the study website, which contains information on eligibility criteria and informed consent and (2) complete a Web-based screening questionnaire to determine eligibility. As part of this process, participants provide their civil registration number, which is a unique, personal 10-digit number assigned to all Danish residents at birth or upon immigration. No incentives are offered to participants, who are followed until they achieve a pregnancy, start fertility treatment, or end of observation (12 cycles). In both studies, enrollment is based on completion of a comprehensive baseline questionnaire, which takes an average of 22 minutes [28].

A substudy, SnartForaldre.dk/Milieu, was introduced to pilot test the feasibility of collecting blood and urine samples to investigate associations between endocrine disruptors and fertility. Participation in the substudy required enrollment in SnartForaeldre.dk and residence in the Aalborg area (population of 205,407 in December 2013) [29].

### Recruitment Methods

During the study period, we employed several online and offline recruitment methods to increase awareness about the study and to encourage enrollment. All advertisements were designed by the same graphics designer and used images, colors, and text phrases identical with or similar to the respective study website to enhance recognition. The most frequently used text was “Planning to get pregnant? Help us find out whether lifestyle influences your ability to become pregnant.”

#### Offline Recruitment Methods

Three offline recruitment methods were used to attract attention to our study: press releases, posters, and flyers.

A total of 6 press releases with various topics were issued; of which, 3 press releases were written and disseminated with the assistance of contracted, external journalists, 1 was handled by study staff alone, and 2 were issued with help from an internal journalist from Aarhus University. We obtained information from media surveillance companies on the numbers of printed and Web-based articles, and radio or TV features that the press releases resulted in.

The costs of obtaining the services of external journalists and a media surveillance company were €2281.29 and €1761.91 for the first 2 press releases, respectively, and €1772.54 for the fourth press release. Issuing the other press releases involved no direct costs as internal institutional resources were used.

**Figure 2:**
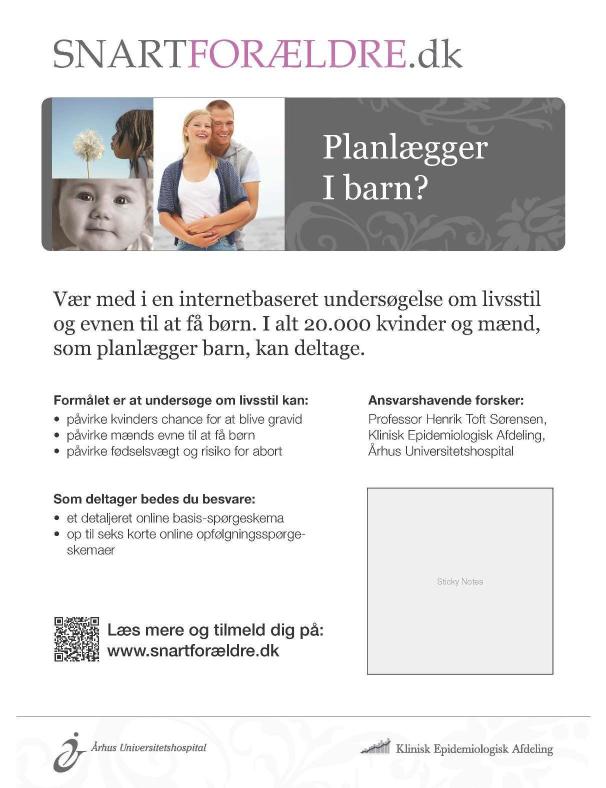
Poster advertising SnartForaeldre.dk.

A small A4-size (210x297 mm) poster with information about the study was designed (Figure 2). Each copy was equipped with a block of 50 post-it notes providing study information. The poster included a quick response (QR) code providing a direct link to the study website when scanned with a smart phone. Study staff and colleagues placed 133 posters on notice boards in libraries, hospital canteens, fitness centers, grocery stores, and other public places. In total, €377.76 was paid for the design of the poster, the QR-code, and the post-it notes and for printing.

**Figure 3:**
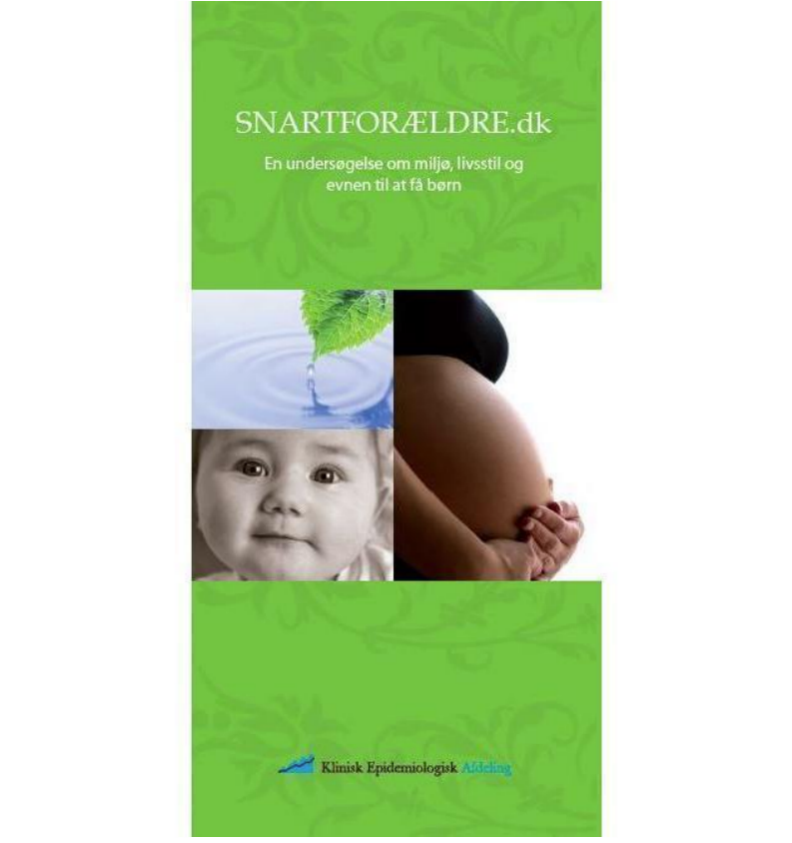
Flyer advertising SnartForaeldre.dk and the substudy SnartForaeldre.dk/Milieu.

Flyers advertising SnartForaeldre.dk, and the substudy, SnartForaeldre.dk/Milieu were distributed to pharmacies, a few stores, and 54 general practitioners’ (GP) offices in the city of Aalborg (Figure 3). Costs for designing the flyer and printing 3500 copies were €604.31.

#### Online Recruitment Methods

Online campaigns, primarily consisting of online advertisements, were placed on 6 different websites: Netdoktor.dk, Minmave.dk, Facebook, Sundhedsguiden.dk, Baby.dk, and Aarhus University (AU). When participants clicked on an advertisement, they were automatically redirected to the study website.

**Figure 4:**
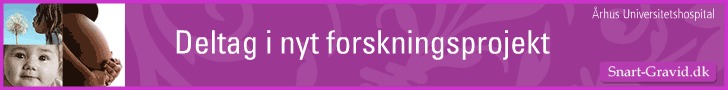
Online advertisement for Snart-Gravid.dk posted on Netdoktor.dk.

**Figure 5:**
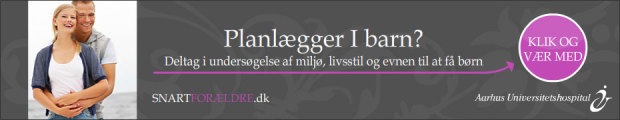
Online advertisement for SnartForaeldre.dk posted on Netdoktor.dk.

Netdoktor.dk is a popular general health website. Two online advertisements of different sizes were posted 2 weeks after the launch of Snart-Gravid.dk (Figure 4). Netdoktor.dk agreed to show the advertisement until it had generated 2500 participants at a fixed price of €3.93 each. For the SnartForaeldre.dk study (Figure 5), Netdoktor.dk committed itself to recruit 10,000 women at a fixed price of €2.99 per enrolled participant. Netdoktor.dk controlled the timing of advertisements to be able to prioritize campaigns by full-price advertisers.

Facebook is a popular social media website. In total, 8 advertisements, lasting 8-10 days each, were posted on Facebook. The advertisements were targeted at potential participants, that is, female, 18-40 years old, speaking Danish, and married or in a relationship. Seven advertisements targeted women living anywhere in Denmark and one targeted only women living in the city of Aalborg.

Advertising on Facebook is managed on the Internet and the price is based on a bidding system. Depending on the number of advertisers aiming at the same target group, the system suggests a price range, within which it is likely that your advertisement will be shown. It is possible to pay per 1000 impressions, that is, the number of times an advertisement is shown on the site, or per click and to choose a daily spending limit. We paid per click and chose a limit of €13.33 per day for 8-10 days at a time. The SnartForaeldre.dk study staff also created a page on Facebook to promote the study. Postings were made by staff and thus incurred no direct costs.

Sundhedsguiden.dk is a general health website. We made an agreement including 100,000 impressions of banner advertisements at an overall fixed price of €1077 over a period of 8 months.

Minmave.dk is a website for pregnant women, that is, women who want to become pregnant and women with infants. We negotiated an agreement covering 5 one-month campaigns, each consisting of 500,000 impressions of banner advertisements, 50,000 pop up/overlay banner impressions within the category “Fertility,” and short texts in 3 electronic newsletters distributed to subscribers. An overall fixed price of €2019 was paid for each of the 5 campaigns.

Baby.dk is a website aimed at parents of infants, pregnant women, and women wanting to become pregnant. We made an agreement including continuous display of a top banner advertisement and a continuous “ownership” picture on a fertility page for 3 months, as well as placement of 2 stories about our study on the website at an overall fixed price of €673.

The Aarhus University Communications Department placed 2 short postings about SnartForaeldre.dk, including links to the study website, on the AU intranet, which reaches approximately 38,000 students and on the AU Facebook page. The postings were free of charge.

### Assessment of Number of Participants Recruited by Each Method

We defined seven categories of mutually exclusive recruitment methods: online advertisements, press releases, posters, flyers, SnartForaeldre homepage, other homepages, and “other.” We used two data sources, electronic tracking and self-reported data, to determine the recruitment method for each participant. The number of participants enrolled as a result of online advertisements was obtained by electronically tracking participants’ click on the advertisements via Uniform Resource Locator (URL) using different URL codes for each online campaign. URL tracking overruled any self-reported response by the participants. Facebook provided detailed statistics regarding the number of impressions and the number of clicks for each advertisement placed on its website. In addition, we calculated a click-through rate, that is, the number of times someone clicked on an advertisement divided by the number of times it was shown, and a conversion rate, that is, the number of people who enrolled after having clicked on the advertisement (and thus registered by its unique URL) divided by the total number of people who clicked on the advertisement.

The remaining participants were categorized according to their response to the following question on the baseline questionnaire: “How did you hear about the present study?” The response options were: “SnartForaeldre website,” “Facebook,” “Netdoktor,” “Other websites or blogs or chatrooms,” “Poster,” “Flyer from my GP,” “Radio,” “Television or teletext,” “Newspapers,” “Weekly magazines,” “Previously participated in study,” and “Word of mouth,” as well as an open-ended “Other.” It was possible to mark multiple response options. Any open-ended response that could be linked unambiguously to an online advertisement was categorized as such.

The number of women who enrolled as a result of press releases was determined by identifying those who, within a period of 2 weeks after a press release, marked at least one of the following responses: “Other homepages,” “Radio,” “TV or teletext,” “Newspapers,” “Weekly magazines,” or “Other magazines,” without marking any other answers. In a sensitivity analysis, we expanded the time period to 4 weeks.

In a subanalysis, we estimated the average number of participants recruited per 30 days for each online recruitment method.

Finally, we excluded women who did not respond to the question “How did you hear about the study?” or who outside a press release window marked more than one answer. The reason for these exclusions was the inability to assign respondents to one specific recruitment category.

### Costs

For each recruitment method, costs per enrolled participant were calculated by dividing the total direct expenses (which did not include salary expenses for study staff) by the number of participants recruited from that method. For the recruitment methods “SnartForaeldre homepage,” “other homepages,” and “other,” costs per enrolled participant were not calculated as these methods were free of charge. All costs are reported in euros, applying exchange rates as of March 2012.

## Results

### Overall Recruitment

**Figure 6:**
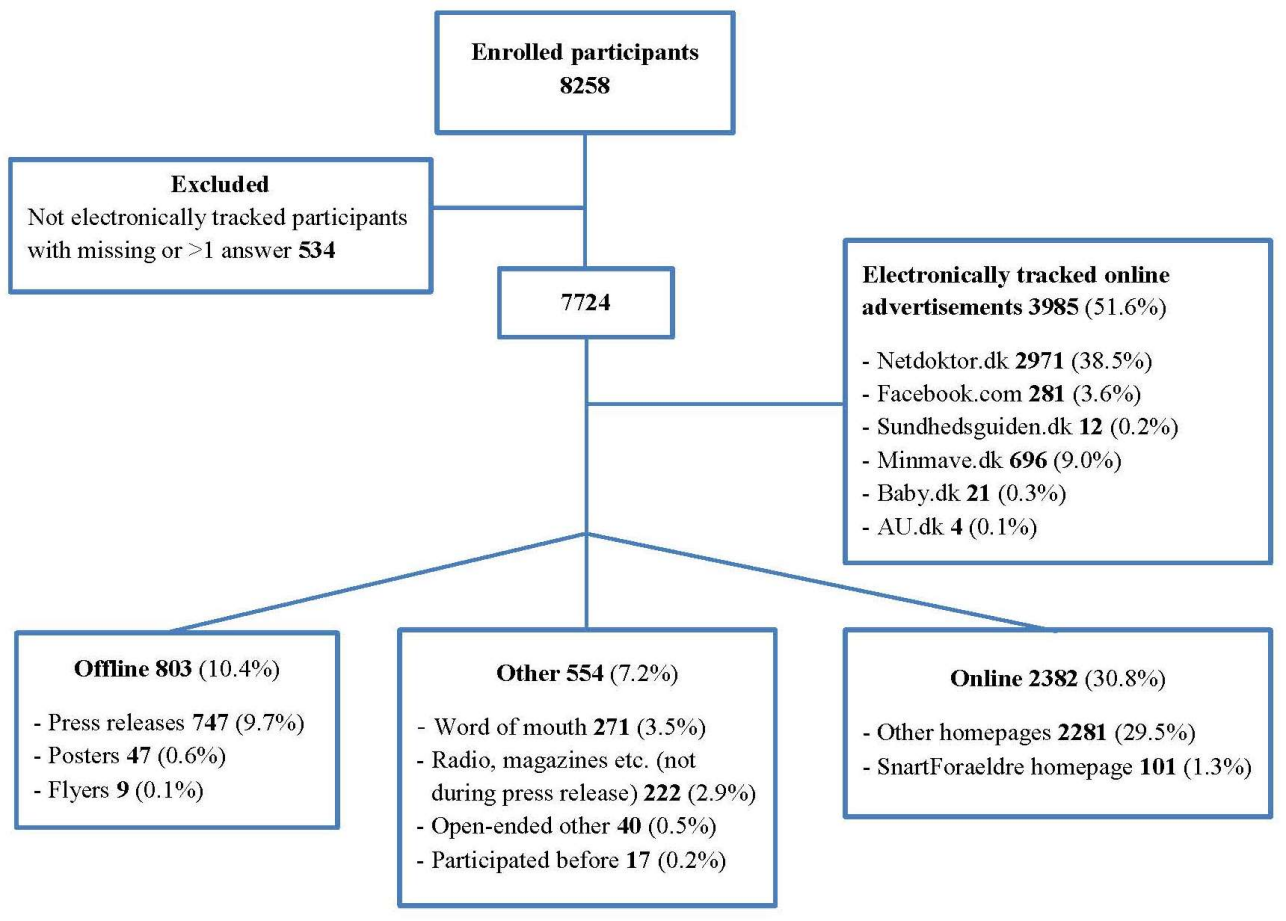
Number of participants by recruitment method.

In total, 8258 participants were recruited for our Web-based cohort study during the study period (Figure 6). We excluded 534 (6.5%) participants from this analysis as they either did not answer the question on how they heard about the study (n=19) or they provided more than one answer (515 respondents, among whom 452 provided two answers, 59 provided three answers, and 4 gave four answers). Thus, the final study population consisted of 7724 participants. Of these, 3985 (51.6%) were recruited by online advertisements, 803 (10.4%) were recruited by offline methods, and 2382 (30.8%) were recruited from two other online methods, that is, “SnartForaeldre homepage” and “other homepages” (see Figure 6). Other methods generated 554 participants (7.2%), of whom 271 (48.9%) learned about the study from word-of-mouth, whereas 222 (40.1%) heard about the study through radio, TV, newspapers, or magazines outside the time window of a press release. Of the 3985 participants recruited from online advertisements, 3866 were tracked electronically and 119 were categorized based on self-reported data.

### Offline Recruitment Methods

For the offline methods, six press releases yielded 747 participants in total. The press releases generated between 8 and 81 articles/features and recruited between 21 and 506 participants at a cost of €0 to €24.28 per participant. Three press releases were issued without external assistance and were not assigned a price (Table 1). The sensitivity analysis in which the time window of the definition of the “press release recruitment” was changed to 4 instead of 2 weeks did not change the cost per participant for the last four press releases. However, for the press release “Launch of Snart-Gravid.dk” the average cost per participant decreased from €4.51 to €3.35 and for the second press release “Funding received to continue study,” the average cost declined from €18.35 to €11.82. Distribution of 133 posters resulted in 47 participants at a cost of €8.04 each, whereas the distribution of 3500 flyers for display led to 9 participants, each costing €67.50 (Table 1). The average cost per participant recruited via offline recruitment methods initiated by study staff was €9.06.

**Table 1:** Costs and number of participants recruited by offline recruitment methods.

Method		Characteristics	Participants N	Average costs per participant €
**Press releases**				
	Launch of Snart-Gravid.dk	35 articles/features	506	4.51
	Funding received to continue study	50 articles/features	96	18.35
	Investigating impact of H1N1 influenza	8 articles/features	28	0.00
	Launch of SnartForaeldre.dk	58 articles/features	73	24.28
	Results: physical activity	84 articles/features	23	0.00
	Results: oral contraceptives	81 articles/features	21	0.00
Posters		133 placed in canteens, supermarkets, and so on	47	8.04
Flyers		3500 distributed to GP^a^ offices, pharmacies, and so on	9	67.50
All offline methods			803	9.06

^a^GP: general practitioners.

### Online Recruitment Methods

Online methods comprised online campaigns including banner advertisements on various websites. A total of 4 years and 7 months of frequent advertisements on Netdoktor.dk generated 2912 participants, each at a fixed price of €3.93 in Snart-Gravid.dk and €2.99 in SnartForaeldre.dk. Factoring in 59 participants who stated that they heard about the study from Netdoktor.dk, but who were not tracked electronically, the costs were €2.74 per participant in SnartForaeldre.dk (Table 2). The average number of participants recruited via Netdoktor.dk per 30 days was 73 in Snart-Gravid.dk and 27 in SnartForaeldre.dk.

**Table 2:** Costs and number of participants recruited by online recruitment methods.

Online advertising campaigns		Characteristics		Participants N			Average costs per participant €	
				Tracked	Tracked per 30 days	Self-reported	Tracked	Tracked and self-reported	
**Netdoktor.dk**								
	Snart-Gravid.dk	One campaign^a^, 31 months		2273	73	0	3.93^a^	N/A^b^
	SnartForældre.dk	One campaign^a^, 24 months		639	27	59	2.99^a^	2.74
Minmave.dk		Five campaigns, 1 month each		689	138	7	14.93	14.78
			October 1, 2012	212		N/A	9.70	N/A
			January 20, 2013	158		N/A	13.02	N/A
			March 1, 2013	142		N/A	14.49	N/A
			May 14, 2013	88		N/A	23.37	N/A
			August 31, 2013	89		N/A	23.11	N/A
Sundhedsguiden.dk		One campaign, 8 months		11	1	1	115.12	105.53
Baby.dk		One campaign, 3 months		19	6	2	45.40	41.08
AU.dk		Postings on AU^c^ intranet and AU Facebook page		4	N/A	0	0.00	N/A
Facebook		Seven nationwide advertisements, 8-10 days each		230	121	50	3.44	2.83
All online methods				3866	53	119	6.22	6.04

^a^Negotiated, fixed price per recruited participant.

^b^N/A: not available.

^c^AU: Aarhus University.

Five one-month campaigns on Minmave.dk resulted in 689 participants at an average cost of €14.93 each. Factoring in 7 participants who stated that they heard about the study from Minmave.dk, but who were not tracked by a URL code, the average cost was €14.78 each. However, the number of recruited participants decreased from 212 participants in the first campaign to 89 in the last campaign. Consequently, the corresponding cost per recruited participant increased from €9.70 to €23.11 in the course of the campaigns (Table 2). On average, 138 participants were recruited from Minmave.dk per 30 days.

An advertisement on Sundhedsguiden.dk yielded 11 participants at €115.12 each over 8 months, whereas a 3-month campaign on Baby.dk generated 19 participants at €45.40 each. When we included participants who reported that they knew about the study from these websites but were not tracked, the cost per participant was €105.53 for Sundhedsguiden.dk and €41.08 for Baby.dk (Table 2).

Seven nationwide advertising periods of 8-10 days on Facebook yielded 230 participants at an average cost of €3.44 each (Table 2). Factoring in 50 participants who stated that they heard about the study from Facebook, but who were not tracked electronically, the average cost per participant was €2.83. It was found that one Facebook advertisement targeted only at women living in Aalborg, that is, a small target population, resulted in 1 participant at the cost of €21.94 (Table 3). In terms of individual nationwide Facebook advertisements, the number of recruited participants varied between 22 and 84 for the first four advertisements, whereas the number was 11 or less for the last three advertisements. The average cost per participant ranged from €1.32 to €22.18. The lowest number of impressions was 16,776 and the highest was 409,129. The click-through rate varied between 0.13% and 1.85%. The conversion rate steadily decreased from 12.07% for the first Facebook advertisement to 1.61% for the last. The average number of participants recruited from Facebook per 30 days was 121.

The average cost per participant recruited via any online recruitment method initiated by study staff was €6.22 for those tracked electronically compared with an average of €9.06 for offline methods.

**Table 3:** Costs and number of participants tracked by Uniform Resource Locator codes in advertisements on Facebook.

Target area	Time	Impressions^a^ n	Clicks^b^ n	Click-through rate^c^ %	Participants n	Conversion rate^d^ %	Average costs per participant €	
Nationwide	March 30 to April 7, 2010	409,129	696	0.17	84	12.07	1.32
Nationwide	June 29 to July 7, 2010	250,324	587	0.23	60	10.22	1.85
Nationwide	November 25 to December 2, 2010	271,176	403	0.15	42	10.42	2.64	
Nationwide	September 22-29, 2011	247,412	324	0.13	22	6.79	5.04
Aalborg	October 9-16, 2011	84,565	39	0.05	1	2.56	21.94
Nationwide	May 3-12, 2012	79,624	157	0.20	6	3.82	16.65
Nationwide	October 31 to November 9, 2013	23,423	344	1.47	11	3.20	12.53
Nationwide	December 21-28, 2013	16,776	311	1.85	5	1.61	22.18
Nationwide, in total		1,297,864	2822	0.22	230	8.15	3.44

^a^The number of times an advertisement was shown.

^b^The number of people who clicked on the advertisement.

^c^The proportion of people who saw the advertisement and clicked on it.

^d^The proportion of people who clicked on the advertisement and were enrolled.

## Discussion

### Principal Findings

In our study population of 7724 pregnancy planners recruited into a prospective cohort study, the majority (6367 participants, 82.4%) were recruited using online recruitment methods. Offline recruitment methods and other methods accounted for 10.4% (803/7724) and 7.2% (554/7724) of participants, respectively. Overall, the average cost per participant was €6.22 for online methods initiated by study staff versus €9.06 for offline methods. Though online methods were the most efficient recruitment tools, costs ranged from €2.74 to €105.53 per participant. Costs per participant for offline methods ranged from €0 (press release issued with institutional assistance, free of charge) to €67.50. We were able to monitor efficiency (the total number of recruited participants), over time for Facebook and Minmave.dk, and found that efficiency decreased while cost per participant increased over time.

### Limitations

We lacked access to information on the number and timing of impressions on Netdoktor.dk. Thus, we were unable to trace any fluctuation in efficiency over time of a prime recruitment method—the advertisements on Netdoktor.dk. We can only speculate whether the efficiency of these advertisements decreased over time as was the case with our advertisements on Facebook and the campaigns on Minmave.dk.

The average number of participants recruited per 30 days from online campaigns varied from 1 (Sundhedsguiden.dk) to 138 (Minmave.dk). Minmave.dk and Facebook yielded the highest average number of participants per 30 days. However, the number of impressions for Netdoktor.dk was unknown and it varied both across and within the other recruitment methods, limiting the comparability of the numbers.

The possible imprecision of the estimated efficiency of press releases is also a concern. The number of recruited participants yielded by this method was defined according to a given time period, which may have been too short. As indicated in our sensitivity analysis, more women may have been recruited from this method than we calculated, and thus, we may have overestimated the average cost per participant.

Another limitation is the possibility that participants who reported that they learned about the study from “Other home pages” (2281 participants, 29.5%) may have seen an online advertisement but not clicked on it. Instead they may have signed up later directly via the study website. Thus, the number of participants recruited by each online advertisement may have been underestimated.

Our exclusion of 515 women who marked more than one answer to the question “How did you hear about the study?” may also be viewed as a study limitation, as it may have led to overestimation of the cost per participant. However, had these women been assigned to two or more categories of recruitment methods, they would have been counted twice and costs would have been underestimated.

### Comparison With Prior Work

#### Online Methods

Our special agreement with Netdoktor.dk involved paying a fixed price of less than €4 per recruited participant and resulted in 2971 participants during the study period. Such an agreement seems unusual. However, Ramo et al [20] reported a similar arrangement with a Web-based sampling service that sent out email invitations to people signed up as volunteers for completing Web-based surveys. They paid a fixed price of US $19.24 (€14.71) per completed survey and achieved 67 in 6 months. Thus, their price was much higher than the price we negotiated with Netdoktor.dk.

Compared with other studies, the overall average cost of €6.22 per participant recruited by online methods in our study is low. Graham et al [21] reported an average cost per registrant of US $209.34 (€160.06) (ranging from US $73.76 (€56.40) to US $4166.67 (€3185.84)) in their study on the effectiveness of advertisements placed on four different Spanish-language websites to recruit Latinos for a Web-based smoking cessation program. Ramo et al [20] paid US $42.77 (€32.70) per participant recruited through an Internet advertising campaign using various social networking and lifestyle-based websites.

For our North American sister study, PRESTO, Wise et al [10] reported costs of US $43.00 to $96.15 (€32.88 to €73.52) per participant for those recruited via online advertisements placed on websites other than Facebook. This agrees with the costs of the less efficient websites used for advertising our study, which cost up to €115 per participant.

The efficiency of advertisements on Facebook varied greatly in our study and decreased over time. As the number of impressions decreased markedly throughout the study period, the advertisements were shown fewer times at the same cost. Interestingly, the click-through rate was considerably higher for the last two advertisements (1.47% and 1.85%) compared with the first six (0.05-0.23%). However, this did not translate into more participants as the conversion rate decreased throughout the study period. Still, the average cost per participant recruited from Facebook advertisements was relatively low at €3.44.

Ramo and Prochaska [13] compared 20 different advertisements placed on Facebook over a 13-month period to recruit 18- to 25-year-olds for a survey on the use of tobacco and other substances. Efficiency varied widely across the advertisements. In addition, both the number of impressions and the number of clicks increased over time, but not concomitantly. Ramo et al paid an average cost of US $4.28 (€3.27) per completed survey, similar to our costs.

In comparison with the average cost of €3.44 per recruited woman via Facebook in our study, costs ranging from €6.73 to €19.48 have been reported in studies on nutrition [14,30], general health [19], smoking cessation [31], and childbirth preferences [7]. Our sister study, PRESTO, and another study by Richiardi et al, with a topic and target group similar to ours, paid US $27.77 (€21) and €25, respectively, per woman recruited from Facebook advertisements [10,17].

In contrast to our results, Thornton et al [12] reported costs of US $1.86 (€1.42) per participant recruited by Facebook advertisements for a survey about tobacco, cannabis and alcohol use, and mental health, whereas Nelson et al [15] paid US $1.36 (€1.04) per participant who completed a Web-based survey on human papillomavirus (HPV) vaccine uptake.

Bearing in mind that the price per click varies according to the Facebook bidding system and the number of advertisers opting for the same target group, costs from the various studies using Facebook depend either on the target country or area, and may not be directly comparable. The criteria by which Facebook advertisements can be tailored to reach specific target populations also vary from one country to another. Wise et al [10] reported that they used “newlyweds” as a target criterion for the PRESTO study in the United States, whereas this criterion was not available on Facebook in Denmark during our study period. Thus, the efficiency of Facebook advertisements may be influenced by how precisely the advertisement can be targeted at potential participants.

Another factor influencing comparability is the use of incentives for enrolling. Announcing incentives before enrollment may make more people want to sign up for the study in question [12,22]. Among other studies using Facebook advertisements, all used incentives [7,10,12-15,19,30], except for one [17]. Apart from the PRESTO study by Wise et al [10], which offered incentives for completing a required number of follow-up questionnaires, incentives were offered for enrollment and thus could have affected people’s inclination to participate. Some studies have reported the use of incentives worth US $15 (€11.46) [30] and US $20 (€15.28) [15] per participant. One could argue that these costs should be included in the overall recruitment cost per participant. However, this would make it more difficult to compare costs directly.

Some studies using online enrollment methods have reported problems with duplicate entries, in particular studies offering incentives [15,20,30]. We did not offer incentives and our study participants had to provide their unique 10-digit civil registration number to enroll, thus ruling out the possibility of multiple entries. The fact that multiple entries into the study was impossible is a major strength of our study and means that the reported number of participants recruited by each method reflects the actual number of distinct eligible individuals responding to the advertisement.

It may be a concern that online recruitment may not yield a sample which is representative of the background population. However, this concern is of relevance in a cross-sectional study that aims to estimate the prevalence of a disease or a risk factor in a given population at a given time. We do not recruit participants to a cross-sectional study, but into a cohort, where we follow our participants until they achieve a pregnancy, start fertility treatment, or end of observation (12 cycles). On the basis of comparisons within the cohort, we estimate associations between various exposures and time to pregnancy. Some differences are likely to exist between our population and all Danish women trying to conceive, but these differences are unlikely to affect the internal comparisons made within our cohort. In addition, in a recent validation study, we showed that the selection bias was not a major concern in the associations under study [25].

The focus of our study on fertility could explain why our recruitment methods proved more efficient compared with some other studies. We had the advantage of an interesting topic—the expectations associated with possible, impending motherhood. Harris et al speculated that the topic of their study on contraception and pregnancy plans motivated women to enroll. Although their study offered incentives, they found that Facebook advertisements not mentioning the incentive, but rather focusing on generating new scientific knowledge to benefit other women, were highly efficient [6].

Similar to other studies that recruit hard-to-reach populations [13,15], we found that online recruitment methods were indeed a viable method to reach our target population.

As was the case with Facebook, the efficiency of the online campaigns on Minmave.dk decreased over time. The average cost per participant thus increased from €9.70 to €23.11. A possible explanation could be saturation of the target population on Minmave.dk. Another explanation could be the increasing tendency toward the use of smaller devices, like tablets and smart phones, for accessing the Internet [18]. However, due to the length of our questionnaire and the comprehensiveness of the questions, it would be cumbersome to complete it on a mobile phone.

#### Offline Methods

The offline methods used in our study were less efficient than the online methods in terms of total number of enrolled participants. In a French Web-based study on nutrition, Kesse-Guyot et al [11] included offline methods to recruit participants and found that only 0.61% and 1.69% of participants learned about the study from posters and flyers, respectively. Our findings are similar, as only 0.61% (47) and 0.12% (9) of our participants reported hearing about the study from posters and flyers, respectively. Kesse-Guyot et al also reported varying effects of their mass-media campaigns, which were launched with a press release and a press conference. They experienced the highest peak in enrollment after the first campaign or press release. This was also the case in our study.

When Wise et al [10] used flyers as a recruitment method, they gained 46 participants (out of 2421 in total) at a cost of US $12 (€9.18) each, whereas postcards led to 15 participants costing US $87 (€66.52) each. Thus, their experience with offline methods is similar to ours in terms of poor efficiency and relatively high costs per participant.

In general, studies employing both offline and online methods to recruit participants for Web-based data collection have found offline methods to be less efficient [6,10,22,23]. This difference may stem from the extra participant effort required by offline methods. After seeing a poster in, for example, the gym, you would have to go online and find the study homepage to enroll. In contrast, when an advertisement is provided on the Internet, enrollment is only a few clicks away [21,23]. It should also be kept in mind that offline methods are more labor-intensive for the research team [7]. This factor was not taken into account in the costs reported in our study. In addition, compared with offline methods, online methods seem more familiar to our young target population, which increasingly conducts daily communications [31].

### Conclusions

We were able to recruit large numbers of women for our Web-based prospective cohort study on fertility. We found that online recruitment methods were superior to offline methods in terms of efficiency (total number of participants enrolled). Both online and offline costs per enrolled participant showed great internal variation. However, given the higher workload associated with offline methods and the lower efficiency, online methods appear the most appealing. The study topic and the use of incentives are likely to influence the efficiency of recruitment methods and the fact that efficiency of online methods may decrease over time suggests that it may be optimal to use multiple online methods.

## Abbreviations

AU: Aarhus University
GP: general practitioner
HPV: human papillomavirus
QR: quick response
URL: Uniform Resource Locator
